# Surfactant Proteins A and D: Trimerized Innate Immunity Proteins with an Affinity for Viral Fusion Proteins

**DOI:** 10.1159/000492974

**Published:** 2018-10-05

**Authors:** Alastair Watson, Maximillian J.S. Phipps, Howard W. Clark, Chris-Kriton Skylaris, Jens Madsen

**Affiliations:** ^a^Child Health, Clinical and Experimental Sciences, Faculty of Medicine, University of Southampton, Southampton General Hospital, Southampton, United Kingdom; ^b^Computational Chemistry, Chemistry, Faculty of Natural and Environmental Sciences, University of Southampton, Southampton, United Kingdom; ^c^Institute for Life Sciences, University of Southampton, Southampton, United Kingdom; ^d^National Institute for Health Research, Southampton Respiratory Biomedical Research Unit, Southampton Centre for Biomedical Research, University Hospital Southampton NHS Foundation Trust, Southampton, United Kingdom

**Keywords:** Collectins, Surfactant proteins, Innate immunity, Virus, Fusion proteins, Structure, Trimeric proteins

## Abstract

Innate recognition of viruses is an essential part of the immune response to viral pathogens. This is integral to the maintenance of healthy lungs, which are free from infection and efficient at gaseous exchange. An important component of innate immunity for identifying viruses is the family of C-type collagen-containing lectins, also known as collectins. These secreted, soluble proteins are pattern recognition receptors (PRRs) which recognise pathogen-associated molecular patterns (PAMPs), including viral glycoproteins. These innate immune proteins are composed of trimerized units which oligomerise into higher-order structures and facilitate the clearance of viral pathogens through multiple mechanisms. Similarly, many viral surface proteins form trimeric configurations, despite not showing primary protein sequence similarities across the virus classes and families to which they belong. In this review, we discuss the role of the lung collectins, i.e., surfactant proteins A and D (SP-A and SP-D) in viral recognition. We focus particularly on the structural similarity and complementarity of these trimeric collectins with the trimeric viral fusion proteins with which, we hypothesise, they have elegantly co-evolved. Recombinant versions of these innate immune proteins may have therapeutic potential in a range of infectious and inflammatory lung diseases including anti-viral therapeutics.

## The Innate Immune Defence of the Upper and Lower Airways

Healthy human adults breathe approximately 15 times a minute, corresponding to thousands of litres of air being inhaled into the lungs every day. Air contains multiple potential hazards for the airways including viruses, bacteria, pollen, and other potentially noxious particles. The area of an unfolded pair of lungs is around 50–75 m^2^ (approx. the size of half a tennis court) [1]. This large surface area needs to be kept as free as possible from infection and inflammation for optimal lung function and thereby efficient gas exchange across the thin alveolar-capillary membrane. When air is inhaled through the nose, nasal hairs and mucus entrap many micro-organisms and particles, preventing their dissemination deeper into the airways. Similarly, micro-organisms and particles inhaled through the mouth are trapped by the mucus in the upper airways and are subsequently removed through the mucociliary escalator. Deeper in the alveolar space, where there is no mucociliary escalator, the area is kept free from hazardous particles and micro-organisms, primarily by the anti-infectious properties of surfactant and the presence of alveolar macrophages. Surfactant is the lower airway fluid lining, which reduces the surface tension of the alveoli to prevent their collapse during breathing. Surfactant is produced by alveolar type II cells and is composed of approximately 90% lipids and 10% proteins. Surfactant is produced in the lower airways, where the majority is degraded by alveolar macrophages or recycled by alveolar type II cells. There is also, however, a movement of surfactant from the lower airways into the upper airways and the mucociliary escalator, which also facilitates lower airway clearance.

## SP-A and SP-D and Their Molecular Structure

There are 4 named surfactant-associated proteins: surfactant proteins A–D (SP-A, SP-B, SP-C, and SP-D). SP-A and SP-D were initially characterised in the 1980s and were originally named SP-35/CP5 and CP4, respectively [2, 3]. The new nomenclature for surfactant-associated proteins was suggested by Dr. Possmayer [4], in 1988, and Persson et al. [5] who proposed that CP4 should be designated SP-D which has been its accepted name since. SP-A and SP-D are hydrophilic calcium-dependent (C-type) lectins with collagenous regions, which thus belong to the group of proteins called collectins [6]. Other proteins in the collectin family include mannose-binding lectin (MBL), the bovine collectins (conglutinin and CL-43) and the recently identified CL-P1, CL-L1, and CL-K1 [7, 8, 9]. In contrast to the collectins, SP-B and SP-C are small hydrophobic proteins. They are involved in the spreading and surface tension-lowering properties of surfactant and are described in more detail elsewhere [10].

**Fig. 1:**
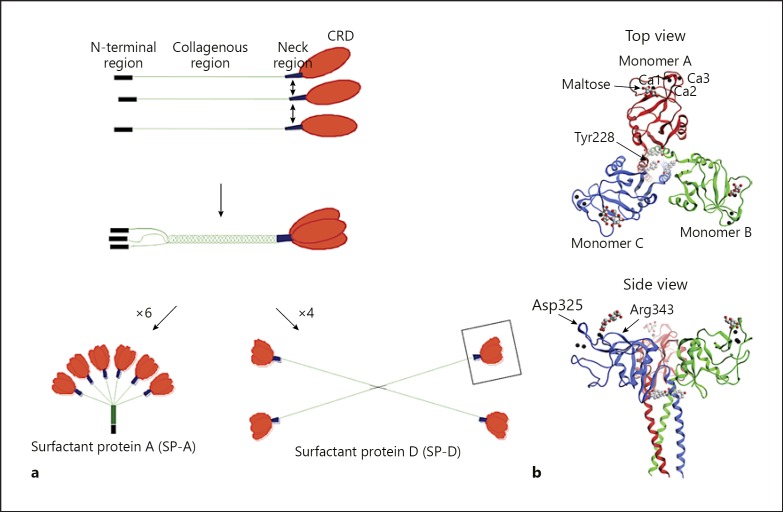
Structure of the collectins. **a** The different oligomeric states of SP-A and SP-D: the carbohydrate recognition domains (CRDs) (orange), neck region (blue), collagenous region (green), and N-terminal domain (black). The box on the higher oligomerised SP-D shows the recombinant fragment of human SP-D (rfhSP-D), which entails 8 Gly-Xaa-Yaa repeats of the collagen-like region, the neck region, and the CRD [20] (this Figure is not to scale). **b** Three-dimensional structures of trimeric rfhSP-D. The 8 Gly-Xaa-Yaa repeats of the collagen-like region, which are part of rfhSP-D, are never identified in the crystallised structure [20, 21, 127]. Top and side view. The 3 calcium ions are shown as black spheres at the lectin-binding site. MOE2014 software with PBD 1PW9 was used to create these figures.

Collectins are oligomerised proteins made up of trimeric units consisting of 3 polypeptide chains. Each chain has a C-terminal carbohydrate recognition domain (CRD), a neck region, a collagenous region and an N-terminal region. The N-terminal region contains cysteine residues, which are important for the higher oligomerisation of the trimeric units (Fig. 1a). For human SP-D, both proline and lysine residues within the collagenous region undergo translational modifications in the form of hydroxylation. Some lysine residues are further O-glycosylated with the disaccharide galactosylglucose [11]. Comparatively, in human SP-A, only proline residues have been reported to be hydroxylated [12], but not lysine residues. Human SP-D also has a glycosylation at serine 72 in the collagenous region whereas SP-A has one at asparagine 187; the glycosylation pattern varies at these residues from simple mono-antennary to multi-antennary glycosylations [11, 12].

The model for collectin biosynthesis is based on in vitro cell culture studies, which investigated the synthesis of rat SP-D with the addition of various protein inhibitors. This model proposes that the CRD regions individually fold, after which trimerisation is initiated within the neck region and forms in a zipper-like fashion along the collagenous tail towards the N-terminal region [13]. Mutagenesis of the 2 cysteine residues in the N-terminal region of SP-D has highlighted their importance for the higher oligomerisation of the molecule, as the mutation of these 2 cysteine residues to serine residues resulted in the SP-D being secreted as trimeric units only [14]. SP-A has only 1 cysteine residue in the N-terminal region, but an isoleucine-lysine-cysteine (IKC) extension at the NH_2_-terminus has been reported for both native rat and human SP-A, thereby showing that some SP-A polypeptide chains in mature SP-A proteins do contain 2 cysteine residues [15].

The exact configuration of the disulphide bridges in the N-terminal regions of SP-A and SP-D has not been determined. However, for CL-43, which is a single trimeric unit, it has been reported that the cysteine residues were linked by inter-chain disulphide bonds between Cys15 in polypeptide chain 1 to Cys15 in polypeptide chain 2, Cys20 in chain 2 to Cys20 in chain 3, and Cys20 in chain 1 to Cys15 in chain 3 [16]. The SP-A protein is assembled from 6 trimeric units and forms an octodecamer which resembles a “bouquet of tulips” (Fig. 1a), and it is similar in structure to the sister collectin MBL as well as C1q, a sub-component of the complement system that does not possess a lectin domain. SP-D is a dodecamer which resembles a cruciform structure (Fig. 1a) but can also assemble into higher oligomeric structures consisting of up to 8 dodecamers, to form “astral bodies” or “stellate multimers”; these make up around 5% of natural SP-D in human lung lavage [17, 18].

The recombinant fragment of human SP-D (rfhSP-D), which consists of the CRD, neck, and 8 Gly-Xaa-Yaa triplets, has been developed and characterised [19]. The crystal structure of the fragment has been resolved, providing an important insight into the structure of the trimeric CRD and neck (Fig. 1b) [20]. The monomers in the trimeric fragment have been named A, B, and C, respectively. These trimerise in a clockwise manner [20, 21]. Human SP-A is comprised of 2 proteins, SP-A1 and SP-A2 [22], and their ratio in the native SP-A protein has been found to vary amongst individuals [23]. The recombinant fragment of human SP-A1 (rfhSP-A), equivalent to the rfhSP-D fragment and containing the CRD, neck, and 8 Gly-Xaa-Yaa triplets, has recently been developed and found to form functional trimeric units [24]. However, the crystal structure of this human SP-A1 molecule is yet to be delineated. The crystal structure of the re combinant fragment of rat SP-A (rfrSP-A) has, however, been determined. This protein showed the same overall trimerised conformation as observed for the rfhSP-D [25].

**Fig. 2:**
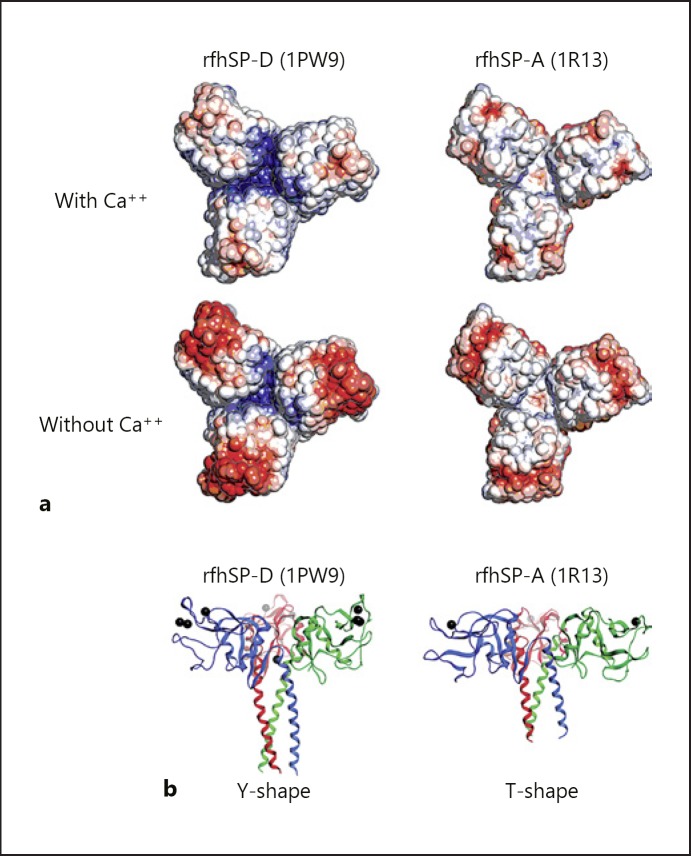
The quaternary structure of the trimeric neck-CRD recombinant fragments of human SP-D (rfhSP-D) and rat SP-A (rfrSP-A). **a** The electrostatic potential of trimeric neck-CRD rfhSP-D (left) and rfrSP-A (right). Isovalues from red (–3.0) to blue (+3.0). PyMOL v1.6.0 and APBS v1.2 were used to create this Figure. **b** Three-dimensional structures of trimeric neck-CRD rfhSP-D (left) and rfrSP-A (right) showing the “Y” shape of SP-D and the “T” shape of SP-A. The 3 calcium ions found for SP-D and 1 for SP-A are shown as black spheres at the lectin-binding site. MOE2014 software was used to create this figure (PDB numbers appear in parentheses).

As mentioned above, the initiation of trimerisation takes place in the neck region. The importance of the SP-D neck region in trimerisation has been illustrated by its ability to drive the trimerisation and stabilisation of thioredoxin, a non-collagenous heterologous protein [26]. The neck region of collectins are triple-stranded α-helical coiled coils, composed of 8 α-helical turns which are formed upon arrangement of the 3 polypeptide chains in parallel. Each polypeptide chain contains a characteristic “a-b-c-d-e-f-g” heptad repeat pattern, whereby amino acid residues “a” and “d” are generally hydrophobic, being Val, Leu, or Phe. These hydrophobic residues form the interior of the α-helical coil and interact with the equivalent regions of the other 2 chains to stabilise the neck [27]. A deviation from symmetry has been observed in the projection of a single tyrosine sidechain (Tyr228) from monomer C into the centre of the coiled coil of rfhSP-D, and the asymmetry of this residue influences the orientation of 1 of the adjacent CRDs (Fig. 1b). The neck region shows a structural continuation with the α-helices of the CRDs and is thought to induce a sharp bend in the SP-D molecule. This further orientates the CRDs in the trimer and provides the ability to recognise and bind ligands, including carbohydrates [20]. Each neck region interacts with the neighbouring CRD in an anti-clockwise direction [21, 25]. The 3 CRDs in rfhSP-D have the same overall fold but are slightly asymmetrical in relation to each other, due to the Tyr228 residue in each of the monomers in the neck region [20, 21] (Fig. 1b). There are no direct interactions between the globular domains, but several contacts between the globular domain of one chain and the coiled-coil region of another domain can be observed. The presence of Lys246 and Lys252 residues in SP-D forms a cavity between the 3 polypeptides, with a positively charged surface. This is different from SP-A where no such charged cavity is observed (Fig. 2a).

The overall fold of the CRD is similar in SP-D, SP-A, and MBL, and has a structurally conserved scaffold. The scaffold constitutes the lower part of the CRD and consists of 3 α-helices and 1 β-sheet, which is stabilised by a disulphide bridge. The actual carbohydrate-binding site forms the upper part of the CRD domain and constitutes a looser structure with a canonical double loop. This carbohydrate-binding site is stabilised by an additional disulphide bridge as well as bound calcium ions. This double loop, which constitutes the lectin-binding site, shows more variation between SP-D, SP-A, and MBL than the lower scaffolding part of the CRD. This most likely reflects the different ligand specificity observed between proteins and animal species [20, 21, 25]. Having an arrangement with the lectin-binding site located at the top of each CRD means that the 3 lectin domains in the trimeric unit are facing upwards and can be viewed as a planar surface with 3 binding sites. This trimeric structure, combined with the relatively short distance of 45–61 Å between the lectin domains, increases their combined affinity via synergy between the 3 sites to constitute a high avidity of binding for repeated carbohydrate structures, as is found on the surface of viruses and bacteria. These repeated structures are rarely found on human cells. Furthermore, the lectin domain's preferences for oligosaccharides found on the surface of micro-organisms enables the lectins to be able to distinguish self from non-self in a relatively simple manner.

## Functions of SP-A and SP-D

**Fig. 3:**
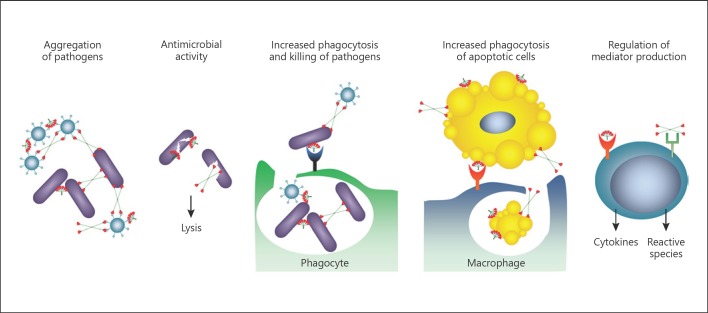
Immune functions of SP-A and SP-D as collectins. They bind to and opsonize viruses, bacteria, worms, allergens, and apoptotic cells. They enhance microbial phagocytosis by aggregating bacteria and viruses. They also possess direct bactericidal effects and potentially bind to a variety of receptors to modulate the expression of immune cell cytokines and inflammatory mediators (adapted with permission from [128]).

The lung collectins SP-A and SP-D have numerous innate immune system functions (Fig. 3), which are reviewed elsewhere in more detail [28]. This review gives a brief overview of the anti-infective and immunomodulatory functions of SP-A and SP-D, and subsequently concentrates on the structure and interaction of these trimeric molecules with viruses with which they have co-evolved. Specifically, we focus on their interaction with the trimeric viral fusion proteins.

The oligomeric collectins contain multiple binding sites and these serve to aggregate microbes such as viruses, bacteria, and fungi [29, 30, 31]. Binding and aggregation of pathogens is calcium-dependent and inhibitable with carbohydrates like maltose, showing that the lectin domain in the CRD is involved in these interactions. SP-A and SP-D proteins bind to and aggregate pathogens but also other non-microbial particles in a similar size range with a repeated surface structure, including pollen [32, 33] and nanoparticles [34, 35]. Similar to the interaction with microbes, the binding to biological non-microbial ligands is calcium-dependent and inhibitable with carbohydrates like maltose, showing the involvement of the CRD in these interactions.

SP-A and SP-D have also been reported to have bactericidal activity by directly inhibiting the growth and viability of several gram-negative bacteria [36]. Both SP-A and SP-D are opsonins and enhance microbial uptake by innate phagocytes, such as macrophages and neutrophils [31, 37, 38]. With the use of flow cytometric and fluo rescent-microscopic assays, SP-A and SP-D have been shown to increase the calcium-dependent neutrophil uptake of both gram-negative and gram-positive bacteria such as *Escherichia coli*, *Streptococcus pneumoniae*, and *Staphylococcus aureus* [37]. In addition to binding to microorganisms, SP-D has been shown to interact with multicellular lung pathogens. It was shown to bind to fucose-containing glycoconjugates which are present on the surface of the helminth *Schistosoma mansoni*, and also to the larval stages of this parasite [39]. Recently, it has also been shown to have a profound effect on the ability to resolve infection with *Nippostrongylus brasiliensis* in a mouse model [40]. Protection was associated with selective binding by the SP-D carbohydrate recognition domain to the lung stage (L4) of the parasite to enhance its killing by alveolar macrophages [40].

SP-A and SP-D are also involved in cellular homeostasis by binding to and promoting the clearance of apoptotic cells [41, 42, 43]. Both SP-A and SP-D have been shown to bind to surface-bound myeloperoxidase [44] as well as DNA and RNA [43, 45, 46], and can therefore bind different ligands found on the surface of apoptotic cells. The binding to DNA is not only part of the clearing mechanism but also has an impact on the clearance of microorganisms via neutrophil extracellular traps (NETs), where it has been shown that SP-D recognises both the short NET fragments and the long DNA structures [47]. Furthermore, it was shown that SP-D could micro-agglutinate the bacterium *Pseudomonas aeruginosa* and allow efficient bacterial trapping by NETs, highlighting the many ways that SP-D facilitates the clearance of micro-organisms via immune cells.

SP-A and SP-D have been shown to bind various receptors on the surface of immune cells [48], and their role in modulating the adaptive immune system, including the function of both antigen-presenting cells and T cells, is becoming increasingly clear. SP-A and SP-D have been found to promote the phagocytic capacity of immature human bone marrow-derived dendritic cells (BMDCs) [49, 50]. However, SP-A was found to regulate the differentiation of dendritic cells (DCs) as measured by their decreased surface expression of CD86 and major histocompatibility complex class II (MHC II) [49, 50]. Contrastingly, using in vitro-generated BMDCs, SP-D was initially found to enhance the surface presentation of a recombinant ovalbumin antigen expressed by *E. coli* [49]. However, when the same research group used ex vivo primary “lung antigen presenting cells”, obtained from mouse lung lavage, SP-D actually decreased the presentation of the ovalbumin antigen [51]. The stimulation of immature BMDCs with SP-A or primary ex vivo “lung antigen presenting cells” with SP-D was also shown to inhibit the stimulation of T cells [52, 53]. This coincides with SP-A and SP-D having previously been shown to inhibit the proliferation of human T cells and the secretion of interleukin (IL)-2 when isolated from peripheral blood [52, 53]. These studies highlight the immunomodulatory functions of SP-A and SP-D to promote phagocytosis and the clearance of pathogens without inducing an inflammatory response. This is important, as inflammation in the lung could damage the thin and delicate alveolar-capillary membrane and negatively impact on gas exchange.

## Calcium-Dependent Binding of Collectins

Each collectin CRD contains a primary calcium site (named Ca1 in SP-D), and 2 additional auxiliary calcium sites have been reported for SP-D and MBL (named Ca2 and Ca3) (Fig. 1b) [21]. An additional auxiliary site has also been reported for SP-A; this was not found in the presence of calcium ions, but with samarium ions, and the position was close to the Ca2 site observed in SP-D [25]. The calcium ions coordinate several charged side chains in the lectin-binding domain and are required for a functional lectin binding (details in [21, 25, 54]). Direct interaction between the lectin domain and a carbohydrate molecule takes place via 2 hydroxyl groups in the carbohydrate unit while the rest of the carbohydrate projects away from the CRD. The key feature of this interaction is the direct ligation of the primary calcium ion (Ca1) with the equatorial 3′ and 4′ hydroxyl groups of a pyranose carbohydrate [21]. This ligation forms an 8-coordinate calcium ion complex with the charged amino acids residues of asparagine (Asn323), glutamic acid (Glu321), Asn341, aspartic acid (Asp342), and Glu329 in the lectin-binding site, explaining the calcium-dependent binding. Human SP-D and rat SP-A have only been crystallised with simple carbohydrates which interact with the primary calcium ion but not the auxiliary one(s). However, it has been suggested that, in vivo, when the collectins are binding to more complex carbohydrates on the surface of microbes, these auxiliary calcium ions might also interact with the ligand(s) [21]. A fourth calcium ion site (Ca4) has been described in the funnel at the bottom between the 3 CRDs of the trimer for human SP-D, but the presence of this was dependent on the concentration of calcium ions used in the cryobuffer; more studies are required to verify this finding [21]. Despite the conservation of ligand-binding residues in collectins, differences in their binding preferences for carbohydrates, both between species and between collectins, are well described [55, 56]. Hence, additional structural features in the flanking region of the lectin-binding domain are influencing the carbohydrate selectivity of the collectins. One example of this is Asp325, which is specific for primates (human and baboon) SP-D, but rat, mouse, cow, and pig have an asparagine residue at this location. This amino acid residue is located at the very ridge of the lectin-binding site (Fig. 1b, side view). This residue does not form hydrogen bonds with the ligand maltose [21]. Binding studies with, for example, *N*-acetyl-mannoseamine (ManNAc) and mannose, showed that a neck-CRD rfhSP-D has a higher affinity for ManNAc than mannose, and computer modelling and docking simulations showed that this difference was probably due to Asp325 directly forming a hydrogen bond with the nitrogen of the NAc group [55]. Another example is Arg343 in primate SP-D, which is located on the other side of the lectin domain from Asp325 (Fig. 1b, side view). This residue is also specific to primates, as rat, mouse, cow, and pig have a lysine residue in this position. Replacing the Arg434 with a lysine (Lys343) in human SP-D was found to have a higher affinity for an *E. coli*-derived LSP ligand than human SP-D. By replacing residue 343 with a lysine in a neck-CRD rfhSP-D (R434K) and an arginine in a neck-CRD rfrSP-A (K343R), it was possible to reverse the affinities of the 2 different splices of SP-D towards the LPS ligand [57]. Hence, the flanking regions of the lectin domain can have an important and direct role in determining the ligand specificity of the molecule.

An important difference between the CRD of rfhSP-D and rfrSP-A is the planar surface of the trimeric unit for ligand recognition. The surface of rfhSP-D has been compared to a “Y-shaped” structure whereas the surface of rfrSP-A is more widely spaced, with the CRD being closer to the neck, and therefore resembling a “T-shaped” structure (Fig. 2b) [25]. The crystal structure of rfhSP-D contains more extensive stabilising interactions than rfrSP-A across the neck-CRD interface [19]. In addition, rfhSP-D contains a funnel formed by the 3 CRDs in the centre of the trimeric rfhSP-D. Here, the 3 Glu232 residues in the trimer, in cooperation with 3 water molecules, coordinate a potential Ca2+ ion (Ca4) into the bottom of this funnel at calcium concentrations > 2 mM [20]. This has been suggested to act as a molecular switch to reduce the neutralizing effect of the Glu232 residues exerted on the Lys246 residues, to alter the charge distribution on the CRD interface, allowing ligand binding. As no crystal structure has been resolved for rfhSP-A yet, it is not known if the same feature is found in human SP-A, but this funnel is absent in rfrSP-A [25].

Another difference between the molecules is the distance between the CRDs. This has been reported to be 51 Å for rfhSP-D [20], 45 Å for human MBL [58], 53 Å for rat MBL [59], and was measured here as 61 Å for rat SP-A. This variation in the average distance between the carbohydrate-binding sites, combined with the quaternary structure of the trimeric unit and the differences in sugar specificity, gives rise to unique and distinctive ligand specificities for each of the proteins. This most likely reflects the co-evolution of the individual collectins and specific microorganisms, since different animal species are exposed to different potentially pathogenic microbes depending on where they live and what they encounter. For example, mice and humans would not be expected to encounter the same pathogens on account of them living below or above ground, respectively.

## Collectins and Viruses: Interactions between Trimeric Molecules

Both SP-A and SP-D have been shown to bind to numerous viruses and the capacity for them to bind specifically to trimerised and glycosylated proteins on the surface of viral capsids is increasingly being understood. This is particularly the case for various enveloped RNA viruses including respiratory syncytial virus (RSV), human immunodeficiency virus (HIV), and influenza A virus (IAV). SP-A and SP-D have co-evolved alongside these viruses to neutralise them through binding to the glycosylated viral-attachment proteins, preventing their binding to the host cell. Alongside preventing the entry of viruses, this interaction can also enhance their aggregation, opsonization, and clearance by phagocytes. The structure of these viral fusion proteins is discussed below, after which the interaction of collectins with different viruses through these fusion proteins is reviewed.

### The Structure of Viral Fusion Proteins

Many enveloped viruses, including all paramyxoviruses discovered to date, express a homotrimeric type I fusion protein called “F protein”. Like other class 1 fusion proteins, such as those of influenza, *Ebola*, and HIV, F proteins have a hydrophobic fusion peptide (FP) and 2 heptad repeat regions (HRA and HRB), are anchored at the surface by a single-pass transmembrane domain (TM), and they contain a C-terminal cytoplasmic tail.

Class 1 fusion proteins are synthesized as a biologically inactive precursor, which are cleaved into their fusogenically active metastable prefusion forms. This cleavage has been suggested to be undertaken by host cell proteases embedded in the cellular membrane, such as the transmembrane protease serine family (recently reviewed in [60]) and mini-plasmin [61] in vivo for influenza and other viruses. Upon activation by the low pH in the late endosomes, the F protein undergoes extensive and irreversible conformational changes. This results in the repositioning of the heptad repeat regions to form a stable 6-helix bundle (6-HB), a process intimately linked to membrane fusion. This model for the conformational change of the F protein is known as “spring theory” [62].

IAV and RSV fusion proteins are made up as a trimer with 3 copies of a single protein. Comparatively, the fusion envelope protein of HIV is made up of 2 non-covalently associated glycoproteins (of 120 kDa and 41 kDa) i.e., gp120 and gp41, respectively (reviewed in [63]).

Another example of fusion proteins which are unrelated in protein sequence but have the same overall trimeric structure are those found on viruses within the herpesvirus family. Herpesviridae is a large family of double-stranded DNA enveloped viruses, which accomplish infection of their host cells through viral membrane fusion with either the plasma membrane or endocytic vesicles. In contrast to the majority of enveloped viruses, which generally require only 1 or 2 viral glycoproteins, herpesvirus infection of cells requires the combined effort of multiple glycoproteins and multiple host receptors, depending on the cell type being infected. This makes it a remarkably complex system. Adding to this complexity, herpesviruses have both pH-dependent and independent entry mechanisms. They share a set of 3 conserved glycoproteins: a heterodimer composed of glycoprotein H (gH), glycoprotein L (gL), and the trimeric class III fusion protein, gB protein [64, 65]. The structure of the postfusion gB protein from *Herpes simplex virus 1* (HSV-1) and Epstein-Barr virus (EBV) has been crystallised. Based on mutation and genetic insertions of fluorescent proteins into the gB protein from EBV and HSV-1, alongside computer models, it has been proposed that the gB protein from both HSV-1 and EBV undergoes a similar prefusion-to-postfusion conformational change to that seen with viral class I proteins. This conformational change mediates the fusion of the viral and cellular membranes [66, 67].

**Fig. 4:**
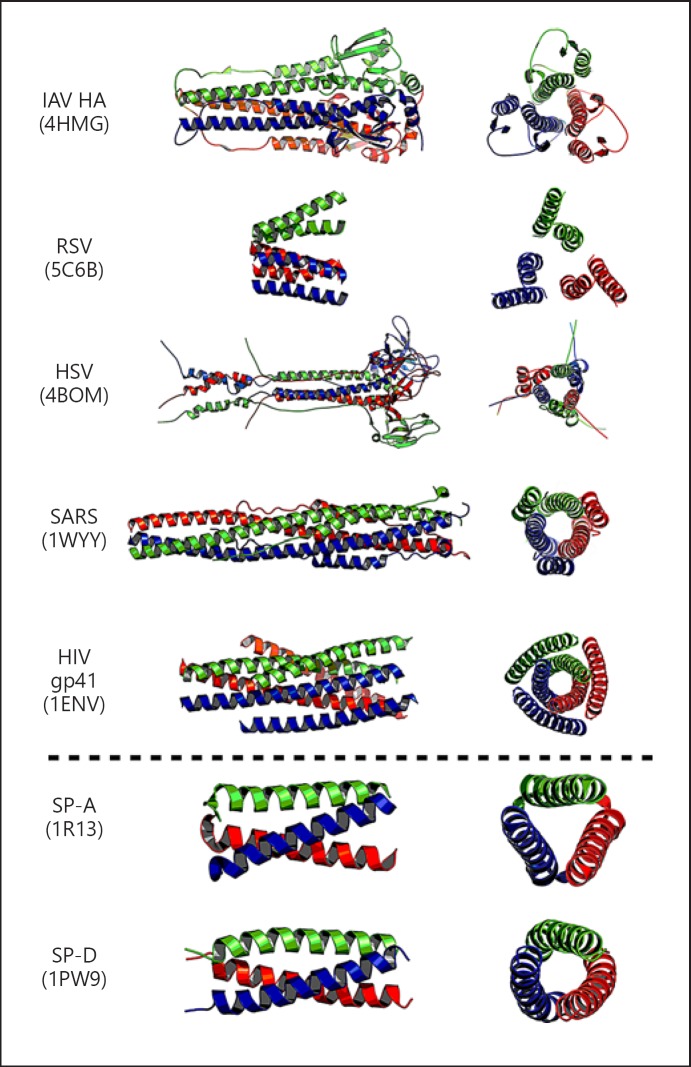
The trimeric configuration of viral fusion proteins and SP-A and SP-D (PDB numbers appear in parentheses). PyMOL v1.6.0 was used to create this figure.

Several viral fusion proteins have been crystallised and their structures identified as either recombinant full-length or fragment forms (Fig. 4). They have a trimeric configuration similar to the trimeric structure of SP-A and SP-D. This similarity in structure and size is intriguing, as SP-A and SP-D could have co-evolved over time to allow their selective binding to these viral surface molecules. This binding can protect the host through various mechanisms. Firstly, direct binding to viral fusion proteins is an important mechanism through which lung collectins can neutralise a virus to directly compete with and prevent it binding to the host cell receptor. Secondly, as the collectins have multiple binding sites per molecule, an important function is to aggregate and clear the virus, either via the gradient of surfactant from the lower to the upper airways through the mucocillary escalator, or via removal by uptake into phagocytes such as macrophages and neutrophils.

### Binding of Collectins to IAV

The binding of SP-A and SP-D to viruses has been studied most extensively with IAV. SP-D binds to high-mannose oligosaccharides in proximity to the sialic acid-binding sites of haemagglutinin (HA) and can therefore neutralise IAV by sterically inhibiting its attachment to host cells [68, 69]. In contrast, IAV binds to sialyated asparagine 187 residue of human SP-A in a calcium-independent manner. This binding occupies the HA-binding site, preventing the binding of IAV to sialylated receptors.

**Fig. 5:**
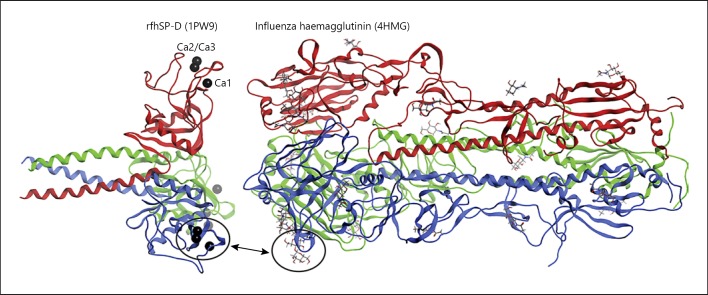
Binding of SP-D to influenza A H3 haemagglutinin. Circles show the lectin domain in 1 of the SP-D monomeric units and the N165 glycosylation site at the tip of 1 of the H3 haemagglutinin molecules shown to be involved in the binding to SP-D. The 3 calcium ions found for SP-D are shown as black spheres at the lectin-binding site (PDB numbers appear in parentheses). MOE2014 software was used to create this figure.

Mouse models deficient for SP-A (SP-A^–/–^) or SP-D (SP-D^–/–^) have shown that both molecules are involved in the clearance of IAV, with deficient mice showing decreased viral clearance of IAV (H3N2 Phil/82 strain) combined with an increased influx of inflammatory cells and production of inflammatory cytokines [70, 71]. The efficiency of SP-D clearance of influenza is proportional to the degree of glycosylation of the HA molecule, with less glycosylated strains showing a greater degree of virulence [70, 72, 73]. SP-D is particularly important in preventing infection by IAV by binding to glycosylations at the very tip of the HA molecule (Fig. 5).

Compared to native (dodecameric) SP-D, which can bind to and agglutinate IAV to modulate infection, the rfhSP-D showed reduced binding to IAV and could not agglutinate the virus or inhibit infection of epithelial cells [74]. Binding studies with a fragment of human SP-D with 2 mutations, Aps325 mutated to an alanine and Arg343 to a valine (D325A + R343V), showed increased binding affinity to HA. The crystal structure confirmed that the mutated SP-D was binding to the glycosylation found at N165 at the very tip of the HA molecule, close to the sialic acid-binding site [75]. This structure was used as a template for in silico modelling of the weaker interaction between HA and a fragment of native human SP-D, formed of just the neck and CRD. This model suggested that this native fragment also binded to the N165 glycan located at the tip of the HA molecule. However, the wild-type and double-mutant SP-D fragments bind to the HA molecule slightly differently. These small differences are enough to change the affinity of the SP-D fragments and the HA molecule, meaning only the double-mutant SP-D fragment neutralises IAV in vitro and in vivo. The importance of the flanking regions was further shown by Crouch et al. [74], who created a new mutant (D325S + R343V) by mutating the Asp325 to serine. This new mutant provided equal to or increased neutralising activity when compared to D325A + R343V against pandemic strains of IAV including strains that normally are fully resistant to inhibition by native SP-D, such as Cal09 H1N1 and other related strains [76].

The HA used in the study by Goh et al. [75] was from the A/Aichi/68/H3N2 IAV, which has only 1 glycan (N165) at the tip of the HA molecule. A recent study used integrated omics and computational glycobiology of HA-resolved structures from 3 different IAV strains with various glycosylations. They found that the wild-type SP-D fragment binded to glycosylations at N165 and also N246 (both located at the very tip of the HA molecule) with 100% efficiency, and they proposed a model of trimeric SP-D binding to the trimeric HA similar to our proposal (Fig. 5) [77]. In 20 simulation models, 18 of these would include either N165 and N246, highlighting that these residues are important for native SP-D being able to bind to and clear the influenza virus [77]. The reassorted X-79 IAV (H3N2 Phils82/A on the outside of the virus particle and A/PR/8/34 on the inside [78]) used in the computational simulation model by Khatri et al. [77] and Xu et al. [78] is normally inhibited by SP-D in wild-type mouse models with normal levels of SP-D in the lungs [79]. SP-D^–/–^ mice with normal levels of SP-A show increased viral titres and cytokine responses when infected with this virus [79]. Mutated X-79 virus, deficient in the glycosylation site at N165 at the tip of the HA1 domain (X-79Δ167), was shown to be 10,000 times more virulent in wild-type mice expressing normal levels of both SP-D and SP-A and had virus titres similar to the mice deficient in SP-D [79]. Furthermore, the concentration of SP-A in the mice did not impact the titres of virus strains X-79 and X-79Δ167, highlighting the different mode of IAV neutralisation for SP-A when compared with SP-D [80].

### Binding of Collectins to RSV

Genetic polymorphisms for SP-A and SP-D genes have been shown to associate with susceptibility to severe RSV infection, highlighting their importance in the immune response to RSV [80]. SP-A^–/–^ and SP-D^–/–^ mice have been shown to have both a decreased capacity for RSV clearance and an increased inflammatory response within the lung [81, 82]. Importantly, rfhSP-A and rfhSP-D have been shown to be effective at neutralising RSV in an in vitro model of bronchial epithelial cells and an in vivo mouse model, respectively [24, 83]. However, it is unclear how SP-A and SP-D interact with RSV. RSV has 2 major surface-exposed glycoproteins, the G protein, which is important for attachment of the virus to the host cell, and the F protein, a type I trimeric fusion protein important for fusion of the virus to the host cell membrane. In one study, SP-A was shown to reduce RSV infection in vitro through binding to the fusion (F) protein of RSV but not to the G protein [84]. However, another study showed that SP-A binds to RSV through the G protein in a calcium-dependent manner, neutralising the virus and enhancing clearance in vivo [85]. Comparatively, rfhSP-D was shown to neutralise RSV through binding to the RSV G protein in a calcium-dependent manner [83]; binding to the RSV F protein in this study was not tested, however. The mechanism by which SP-A and SP-D bind to RSV remains unclear and requires further elucidation, but it may be dependent on the viral strain and viral protein glycosylations. However, given the efficacy of both rfhSP-A and rfhSP-D in neutralising RSV [24, 83] and that the RSV G protein has been reported to not be essential for RSV infection [86], it seems likely that neutralisation by SP-A and SP-D could occur through binding to the RSV fusion protein. The RSV fusion protein is also a trimeric protein with a coiled-coil conformation [87, 88]. The mechanism of the potential binding of SP-A and SP-D to F proteins of different RSV strains now needs to be elucidated.

### Binding of Collectins to HIV

The HIV glycoprotein (gp)120 is essential for virus entry into cells and is the primary target for binding HIV by various C-type lectins such as MBL and DC-SIGN [89, 90]. More recently, the capacity for SP-A and SP-D to bind gp120 has also become clear [91, 92, 93]. The binding of SP-A and SP-D was shown to be inhibitable by EDTA as well as various hexoses, illustrating the interaction of SP-A and SP-D to likely be through the CRD binding to the high-mannose structures of gp120. The distance across the top of the oligomeric structure of gp120 (covered with multiple glycosylations) from one glycan tip to another was estimated to be 110 Å, compared to the 51-Å distance between the binding sites of SP-D [20, 94]. Computer modelling of trimerized gp120 showed the CD4-binding site to be located predominantly on the side of the molecule and not close to the glycans located on the top of the trimerized molecule [94]. This, along with the reported binding of gp120 by SP-D not inhibiting the binding of CD4, indicates both that SP-D may bind to the glycosylations on the top of the trimer and that the binding site of SP-D may not overlap with that of CD4. A contradictory report has, however, suggested SP-D to bind to the conserved N-linked glycosylation sites, Asn234 and Asn276, which are proximal to the CD4-binding site; this binding was shown to impact on the binding of CD4 to gp120 [95]. Again, differences between studies may be due to the use of different viral strains, or viruses from different sources which may have different surface protein glycosylations.

SP-A binding has been shown to inhibit CD4 binding by gp120, highlighting the potential role of SP-A in the neutralisation of HIV by blocking CD4-mediated fusion [92]. SP-D binding to gp120 was, however, shown to prevent the binding of DC-SIGN to gp120, which was also shown in some degree for SP-A [91, 92]. SP-A and SP-D did not prevent the binding of cyanovirin to gp120 [91, 92], highlighting that the binding of SP-A and SP-D to gp120 is likely similar to the DC-SIGN-gp120 interaction, where no single high-mannose N-linked glycosylation site is responsible for the binding of DC-SIGN. This contrasts with cyanovirin, which neutralizes through targeting a specific set of N-linked glycosylations. Interestingly, SP-D has also been reported to bind gp41 which is essential for formation of the gp120 trimer and assists in viral membrane fusion to the cell membrane [95].

As both SP-A and SP-D have been identified in the female genito-urinary tract, the binding of these proteins to gp120 could be important in HIV infection (as the primary site of infection) and in the lung (as a common HIV reservoir site) [96, 97]. This binding has been shown to neutralise HIV and prevent direct infection of a CD4+ T cell line, PM1. However, SP-A and SP-D enhanced the infection of immature monocyte-derived DCs (IMDDCs) and transfer to the CD4+ T cell line, upon co-culture [91, 92]. The mechanism by which collectins enhance this transfer, however, has not been elucidated. This enhanced uptake could either be through the oligomeric structures of SP-A and SP-D allowing the aggregation and enhanced uptake of HIV by the DCs, or through the interaction of the N-terminal domain with a receptor on the DCs to provide a route of entry for HIV into the host cell. A rfhSP-D molecule lacking the N-terminal domain has also been shown to neutralise HIV in vitro [95]. Its interaction with DCs was not studied, however.

Further work investigating the interaction of rfhSP-A and rfhSP-D with HIV compared to the native proteins is important to elucidate the roles these proteins play in HIV infection. These prospective studies could identify whether a fragment lacking the N-terminal region has the capacity for neutralisation, whilst lacking the capacity to enhance transfer from DCs to CD4+ T cells which is likely mediated through the N-terminal domain. Should this be the case, these functional recombinant fragments could have the therapeutic potential to prevent the infection and spread of HIV.

### Binding of Collectins to Other Enveloped Viruses

SP-D has also been shown to bind to the severe acute respiratory syndrome (SARS) virus [98]. SARS, also an enveloped virus, belongs to the family of Coronaviridae which commonly cause infections in both humans and animals. There have been 2 self-limiting SARS outbreaks, in 2002 and 2004, which resulted in a highly contagious and potentially life-threatening form of pneumonia. The SARS virus has a trimerised protein called the Spike protein or S-protein, which is a class I virus fusion protein. SP-D was found to bind to recombinant trimeric proteins, and the binding was calcium-dependent and inhibited by maltose, displaying the characteristics of a classical C-type lectin-carbohydrate interaction [98]. The S-protein did not interact with purified MBL, showing that the interaction was specific to SP-D and highlighting that there are ligand differences between the collectins.

SP-A has also been shown to bind to HSV [99, 100], which has trimerised surface proteins similar to HA on IAV. The interaction between SP-A and this virus has not been studied in detail but was found to be dependent on the glycosylation status of the SP-A protein itself, thereby resembling the interaction described between SP-A and IAV.

## Other Trimeric Innate Immunity Proteins

Trimerisation is an effective way of generating proteins with a high avidity towards ligands. The individual subunits in trimeric innate immune proteins all have a low affinity towards their ligands but a high avidity due to the 3 binding sites per unit. The adaptive immune system is thought to have developed after the innate immune system (reviewed in [101]). Due to the high affinity and specific nature of antibodies and the T cell receptor, it seems that 2 binding sites are sufficient for the adaptive immune response, compared with 3 in the innate immune system.

Other innate immunity proteins have a structure similar to the collectins, composed of trimeric units with a collagenous region. Both C1q and ficolins are formed from trimerised units oligomerised into a “bouquet”-like structure, similar to SP-A and MBL. C1q is made up of 3 sub-chains, C1qA, B, and C, and consists of 2 homotrimers of each subunit. The globular heads are not lectin domains but globular domains with an affinity for the constant region (Fc) of immune complexed immunoglobulin (Ig)M and IgG. Three human ficolins have been described: L-ficolin [102], M-ficolin [103, 104], and H-ficolin [105]. The structure of their trimeric units is similar to the collectins. However, while the collectins have a C-type lectin domain, the ficolins have a fibrinogen-like domain [106, 107] which binds to specific pathogen-associated carbohydrates such as GlcNAc and N-acetylgalactosamine (GalNAc) [105, 108, 109]. H-ficolin also binds to GalNAc and D-fucose, but not to mannose and lactose [105, 109]. L-ficolin and H-ficolin are expressed in hepatocytes and found in the serum [107, 110]. However, similarly to SP-A and SP-D, H-ficolin is also found in bronchoalveolar lavage after expression by alveolar type II cells and bronchial epithelial cells [110]. M-ficolin is mainly associated with the surface of peripheral blood leukocytes but has also been found to be synthesised by alveolar type II cells [104, 108].

L-ficolin has been reported to bind both HA and neuraminidase glycoproteins and inhibit IAV infection in vitro and in vivo [111]. L-ficolin has also been shown to interact with N-glycans of the enveloped *Hepatitis C virus* (HCV) envelope glycoproteins, E1 and E2, leading to complement activation [112]. The HCV E1 protein has also been found to be a trimerised protein, and a model for a trimerised E1E2 surface molecule, similar to gp120 and gp41 in HIV, has been suggested [113]. M-ficolin/ficolin-1 has also been shown to interact with the surface protein of the *Ebola virus* [114]. The *Ebola* surface protein is also a glycosylated and trimerised protein [115], like the other viral surface proteins already described here. Hence, although this has not been tested, it is likely that SP-A and/or SP-D could interact with this protein as well.

Similar to the collectins, the trimerised innate immunity proteins described above also bind to bacteria, pollen, and other potentially noxious ligands as well as removing dying or dead cells and debris. These multiple functions are essential for keeping the lungs free from infection, whilst balancing the immune system to avoid excessive inflammation or autoimmune responses [116].

## Summary and Future Perspectives

SP-A and SP-D are an essential part of the innate immune system of the lung. Numerous in vitro and in vivo experiments have shown that SP-A and SP-D are implicated in the innate clearance of not only viral infections but also fungi and gram-negative and gram-positive bacterial (reviewed in [28]). These 2 proteins are also involved in the clearance of dying and apoptotic cells [42, 43], and have key immunomodulatory effects on DCs and T cells [51, 52, 53]. These functions are thought to be important in applying a brake to the adaptive immune system, to allow efficient clearance of micro-organisms by the innate immune system whilst maintaining a non-inflamed environment within the lungs. The role of SP-A and SP-D in keeping the lung in a hypo-responsive state is key, as aberrant inflammation can quickly impact on the vital gas exchange of the lungs via the thin alveolar-capillary membrane.

The trimeric structure is a simple and elegant way of achieving a high avidity towards a ligand, and viruses too have exploited this feature. In order for the host to defend itself against these viruses, the innate defence proteins have co-evolved over time. However, the trimeric structure of SP-A and SP-D is not only an advantage for binding to viral proteins but also for interacting with other pathogens with a repetitive surface structure, like bacteria [29, 37]. The repetitive structure of particles like pollen may also have played an important role in the evolutionary development of the collectins [32, 33]. The key advantage of these innate immune defence proteins is their broad selectivity, allowing them to recognise an array of rapidly evolving pathogens including viruses, often through binding to the viral fusion proteins. These trimeric collectins have elegantly evolved to recognise and competitively bind the structurally similar trimeric viral fusion proteins. This prevents their attachment to the host cell, neutralises the virus and enhances its clearance from the body, whilst modulating the adaptive immune system [70, 83].

With the invention of rfhSP-A and rfhSP-D, alongside new innovative ways of producing these proteins, it seems feasible that these naturally occurring defence proteins may have applications as anti-infective/anti-inflammatory therapeutics [24, 109]. Due to the ease of their production, yield, and capacity to be stored and administered in saline as trimers [24, 117], the development of these recombinant fragments appears more viable than the production of full-length proteins. Mutated forms of neck-CRD rfhSP-D with increased affinities towards certain ligands could now be developed. This has already been seen, e.g., in the form of mutated SP-D with an increased avidity of binding to influenza virus and neutralisation [74]. The potential exists, therefore, to design different forms of recombinant collectin fragments for different diseases. This mentioned, one has to bear in mind that changing the sequence of a native protein could cause immunoreactivity and potentially be detrimental to the patient. Asp325 and Arg343 residues are both located at the ridge of the lectin domain (Fig. 1b, sideview) and are specific to human SP-D. This region could potentially be an “immunological hotspot” for the development of antibodies against mutant forms of human SP-D. Indeed, in monoclonal antibodies raised against human SP-D, Asp325 has been identified as a key antigenic epitope [118]. More research is required to evaluate whether it would be more beneficial to use recombinant native forms or mutated forms of the collectins for therapeutic purposes in the future.

The development of new clinical treatments against infectious diseases is critical, particularly at this point in time when both bacterial and viral strains resistant to current treatment options are on the increase [119, 120, 121]. The dual function of these collectins could be of particular advantage when considering therapeutics for inflammatory lung diseases, such as severe asthma or chronic obstructive pulmonary disease, where there is a deficiency in the level of SP-D in the lungs [122, 123]. Notably, these diseases are characterised by chronic inflammation with periodic exacerbations which are often triggered by viruses.

Premature neonates who develop neonatal chronic lung disease have also been shown to have an SP-D deficiency [122, 123]. Supplementation with rfhSP-D as an anti-inflammatory agent could both prevent neonatal chronic lung disease and reduce the risk of infection in these susceptible babies. Exogenous SP-D has already been shown to reduce ventilation-induced inflammation [124]. Furthermore, in a preterm baboon neonate model, it was found that the amounts of SP-A and SP-D in the lavage fluid were indicators of the risk of infection in the evolution of neonatal chronic lung disease [125].

SP-A and SP-D are [124] innate immune proteins which are structurally similar to the viral fusion proteins that they selectively bind. These natural molecules have the potential to be exploited for the development of novel, anti-infective, and immune-modulatory therapeutics.

## Disclosure Statement

The authors have no conflict of interest to declare. H.W.C. and J.M. would like to thank the Medical Research Council for the funding of A.W. through the Doctoral Training Programme at the University of Southampton. C.-K.S. and M.J.S.P. would like to thank the Biotechnology and Biological Sciences Research Council and Boehringer Ingelheim for an industrial CASE (Collaborative Awards in Science and Engineering) PhD studentship (BBSRC grant ref.: BB/I015922/1).
